# Supplementary material for: CoCoA-diff: counterfactual inference for single-cell gene expression analysis
Source: Genome Biol. 2021 Aug 17;22:228. doi: 10.1186/s13059-021-02438-4 (PMC8369635; doi:10.1186/s13059-021-02438-4)
Supplement: Supplementary file 1 — Additional file 1: Figs. S1 to S10. with the figure legend texts. [file 13059_2021_2438_MOESM1_ESM.pdf]

# CoCoA-diff: Counterfactual inference for single-cell gene expression analysis

13:23:26, Jul 15, 2021


## Author names and affiliations

Yongjin P Park[1,2], Manolis Kellis[3,4]

1. Department of Pathology and Laboratory Medicine, Department of Statistics, University of British Columbia, Vancouver, BC, Canada
2. Department of Molecular Oncology, BC Cancer, Vancouver, BC, Canada
3. Computer Science and Artificial Intelligence Laboratory, Massachusetts Institute of Technology, Cambridge, MA, United States of America
4. Broad Institute of MIT and Harvard, Cambridge, MA, United States of America

Contact:

- Yongjin P. Park: `ypp@stat.ubc.ca`
- Manolis Kellis: `manoli@mit.edu`

# Supplementary Figures

## Fig S1.

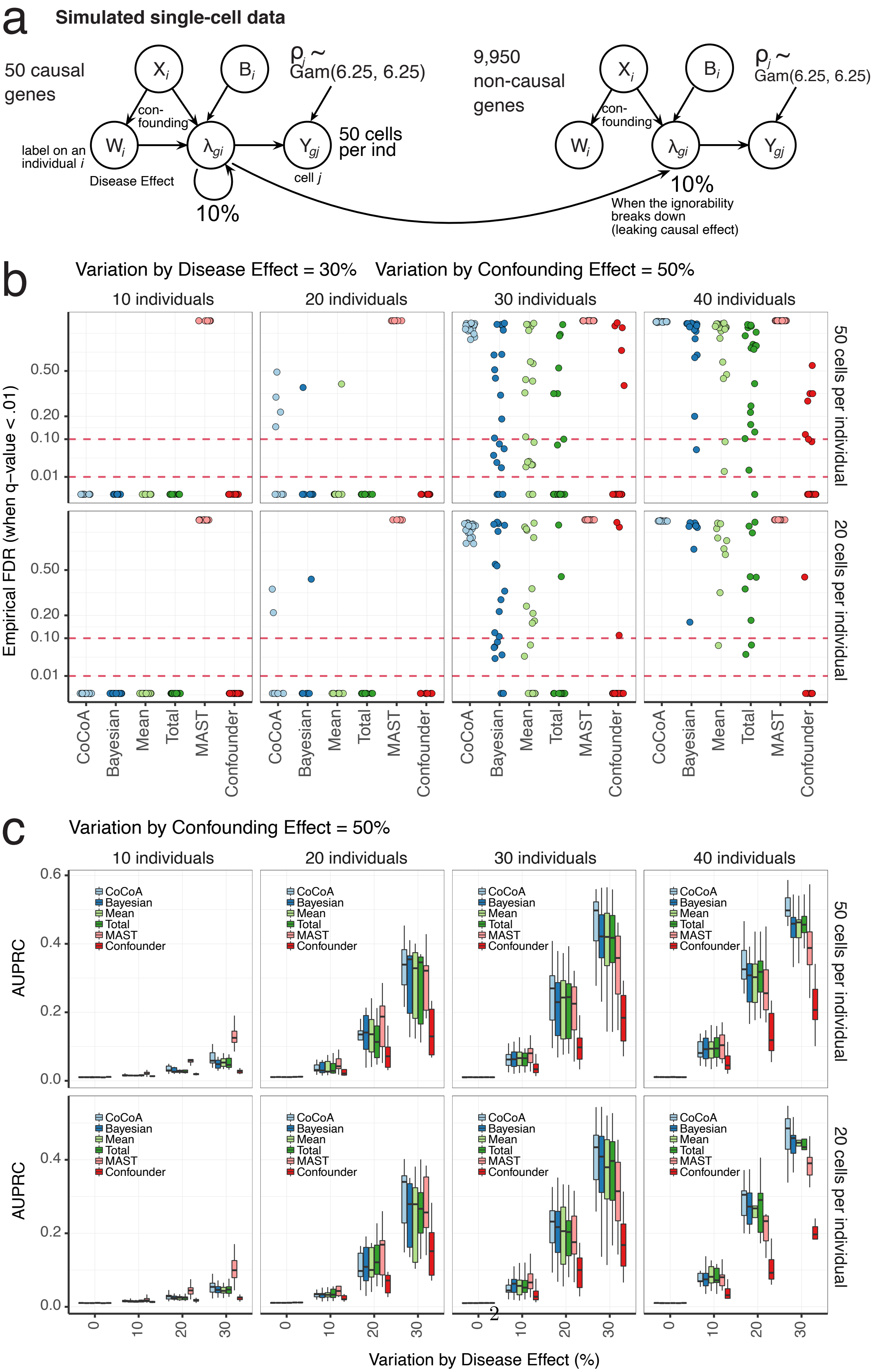

**Simulation experiments with the invalidating collider bias.**

**(a)** Data generation scheme for simulation experiments. We simulate 50 causal and 9,950 non-causal genes with or without disease-causing mechanisms (an edge between $W$ and $\lambda$). $W_i$: disease label assignment for an individual $i$. $X_i$: confounding effects for an individual $i$. $\lambda_{gi}$: unobserved gene expression for a gene $g$ of an individual $i$ as a function of $X$ and $W$. $Y_{gj}$: realization of cell-level gene expression of a gene $g$ with a cell $j$-specific sequencing depth $\rho_j$ (stochastically sampled from Gamma distribution). Here, we simulated total five covariates consisting of confounding ($X$) and batch effect variables ($B$).

**(b)** Empirical false discovery rates of the differential expression methods when there were no confounding effect, but the 30% of individual-level expression variation is attributed to the disease effect ($W \to \lambda$; $\sigma^2_{W\to Y}$) on 50 causal genes. *Y-axis*: empirical false discovery rate, the frequency of the non-causal among genes with the estimated q-value below 0.01.

**(c)** Simulation results when all the five covariates are confounding disease label assignment and gene expression values, accounting for 50% of mean expression variation ($\sigma^2_{X,B\to Y}$). Different subpanels correspond to different configurations of the number of individuals and cells per individual. *Y-axis* (AUPRC): area under precision recall curve (numerically integrated by `DescTool`[1] implemented in `R`); *x-axis*: the proportion of variation contributed by the disease label ($\sigma^2_{W\to Y}$). The following methods were considered: *CoCoA*: Wilcoxon's ranksum test using individual-specific confounder-adjusted gene expression values $\delta_{gi}$ (the step 3 of Fig. 1c); *Total*: pseudo-bulk expression aggregated within each individual; *Bayesian*: Bayesian estimate of pseudo-bulk expression averaged over cells within each individual; *Mean*: pseudo-bulk expression averaged over cells within each individual; *MAST*: Model-based Analysis of Single-cell Transcriptomics[2] implemented in `R` (cell-level differential expression analysis); *Confoudner*: the estimated confounding effect $\mu_{gi}$ (the step 2 of Fig. 1c).

**Fig S2.**

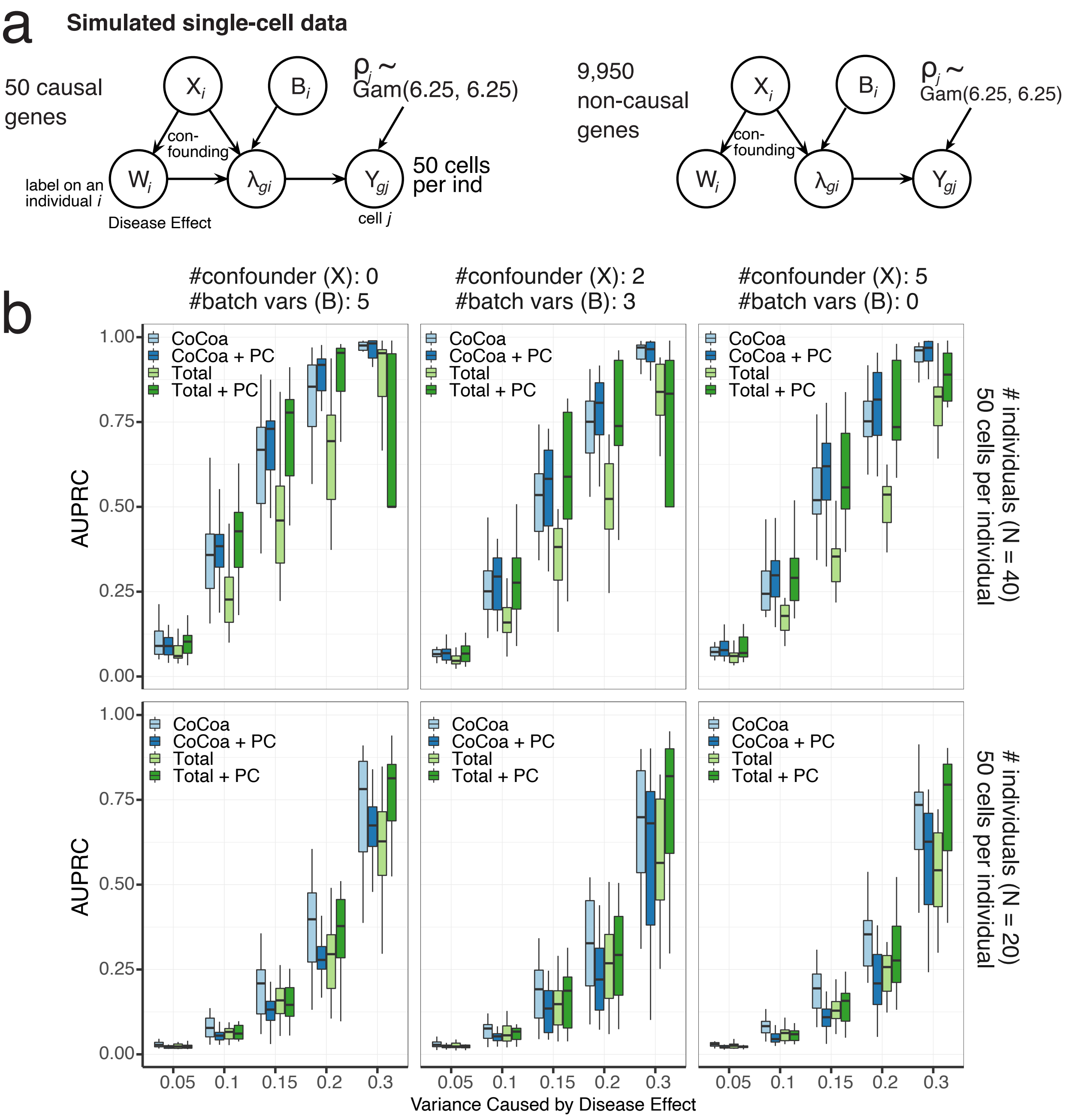


**Simulation experiments with two types of covariates–confounding factors and batch effects.**

**(a)** Data generation scheme for simulation experiments. We simulate 50 causal and 9,950 non-causal genes with or without disease-causing mechanisms (an edge between $W$ and $\lambda$). $W_i$: disease label assignment for an individual $i$. $X_i$: confounding effects for an individual $i$. $\lambda_{gi}$: unobserved gene expression for a gene $g$ of an individual $i$ as a function of $X$ and $W$. $Y_{gj}$: realization of cell-level gene expression of a gene $g$ with a cell $j$-specific sequencing depth $\rho_j$ (stochastically sampled from Gamma distribution). Here, we simulated

total five covariates consisting of confounding ($X$) and batch effect variables ($B$).

**(b)** Simulation results with different numbers of confounding factors and batch effect variables (horizontal subpanels) and different number of individuals (vertical subpanels). *Y-axis* (AUPRC): area under precision recall curve (numerically integrated by `DescTool`[1] implemented in `R`); *x-axis*: the proportion of variation contributed by the disease label ($\sigma^2_{W \to Y}$). The following methods were considered: *CoCoA*: Wilcoxon's ranksum test using individual-specific confounder-adjusted gene expression values $\delta_{gi}$ (the step 3 of Fig. 1c); *Total*: pseudo-bulk expression aggregated within each individual; *CoCoA + PC*: CoCoA followed by PCA on the resulting gene by individual matrix, where the PCs were selected if they are not correlated with the disease labels; *Total + PC*: pseudo-bulk expression followed by PCA, where the PCs were selected if they are not correlated with the disease labels.

## Fig S3.

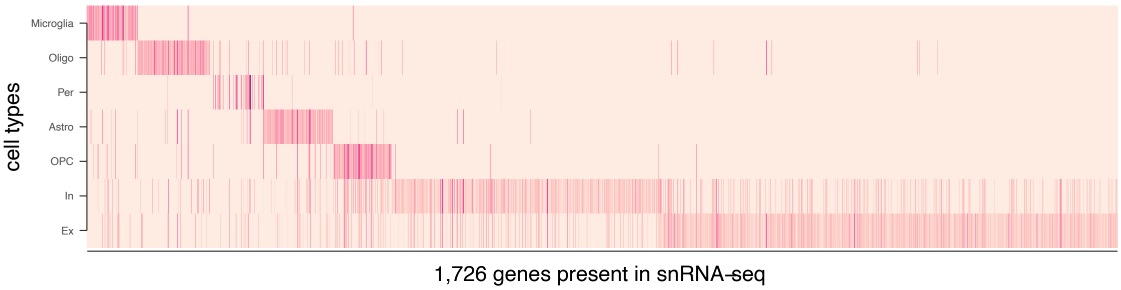


**Average cell-type-specific profiles of 1,726 marker genes**

## Fig S4.

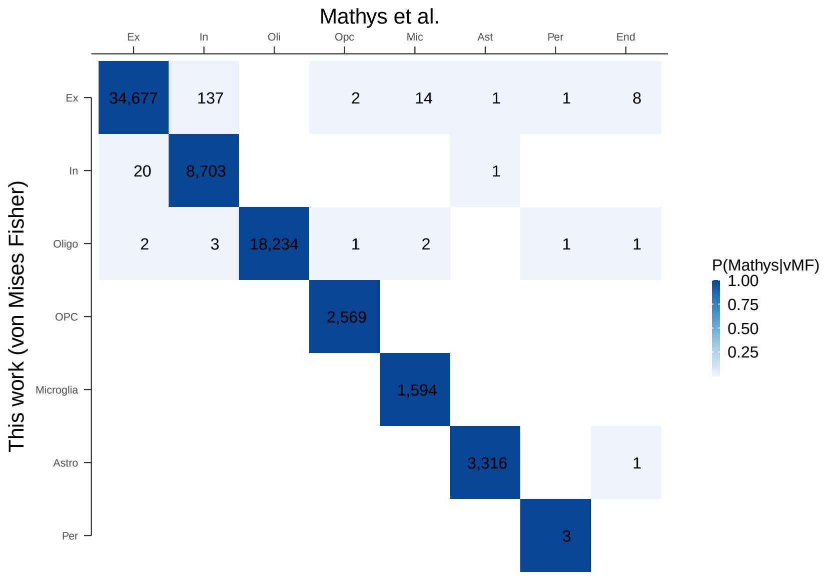


**Correspondence with Mathys et al.[3]**

**Fig S5.**

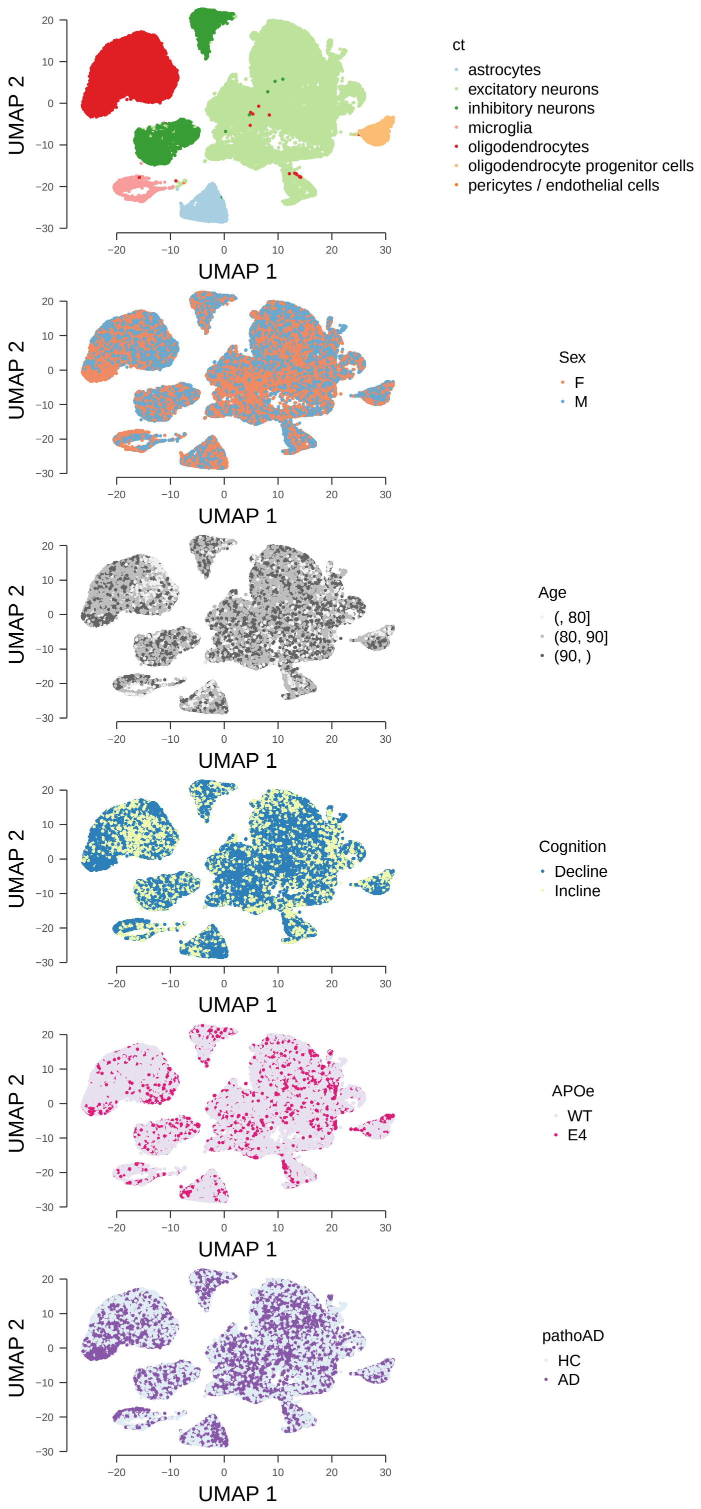


**The annotations of the major neuronal and glial cell types are not biased by known biological variables.**

**Fig S6.**

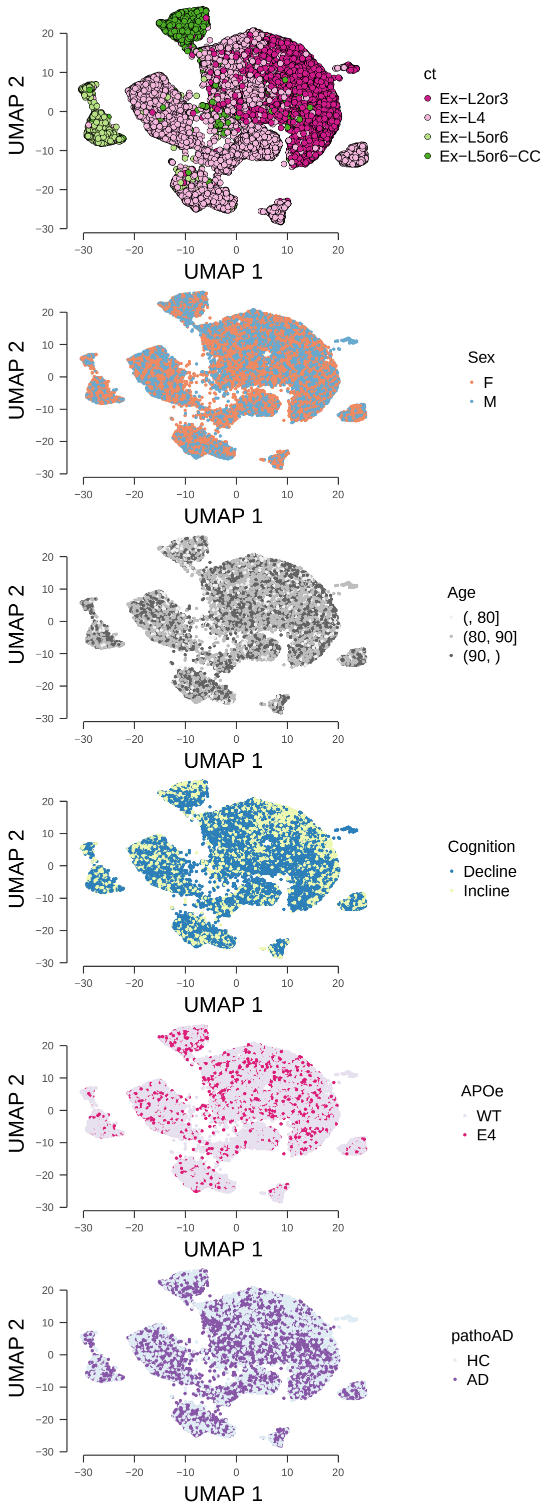


**The annotations of the excitatory neuron types are not biased by known biological variables.**

**Fig S7.**

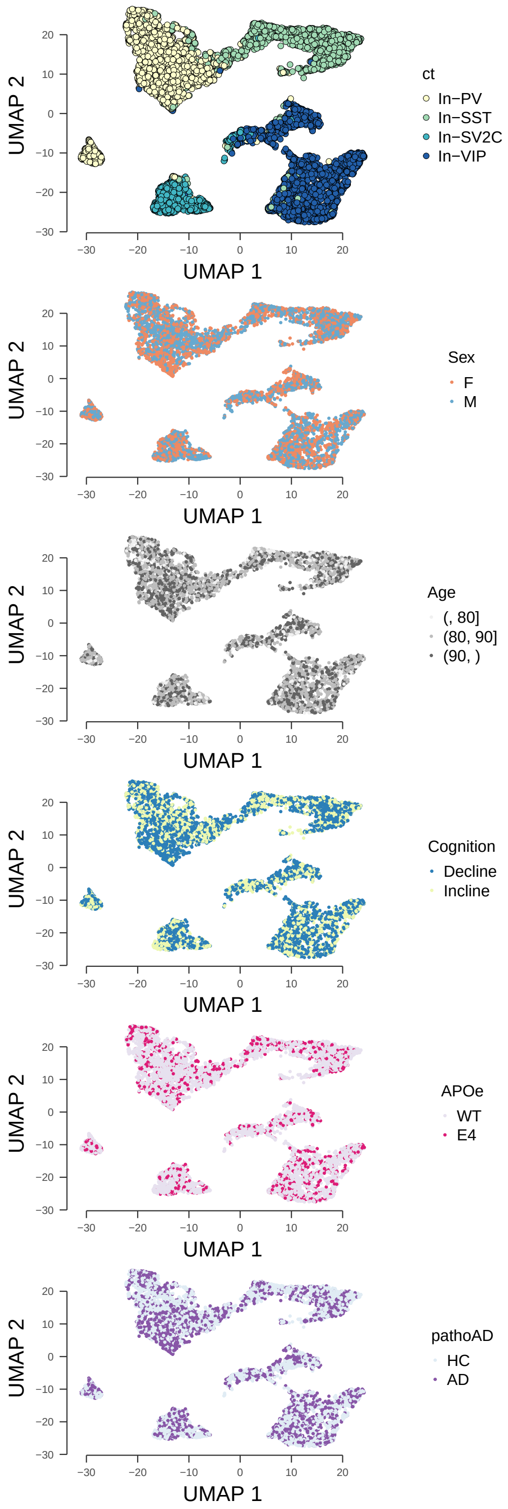


**The annotations of the inhibitory neuron types are not biased by known biological variables.**

**Fig S8.**

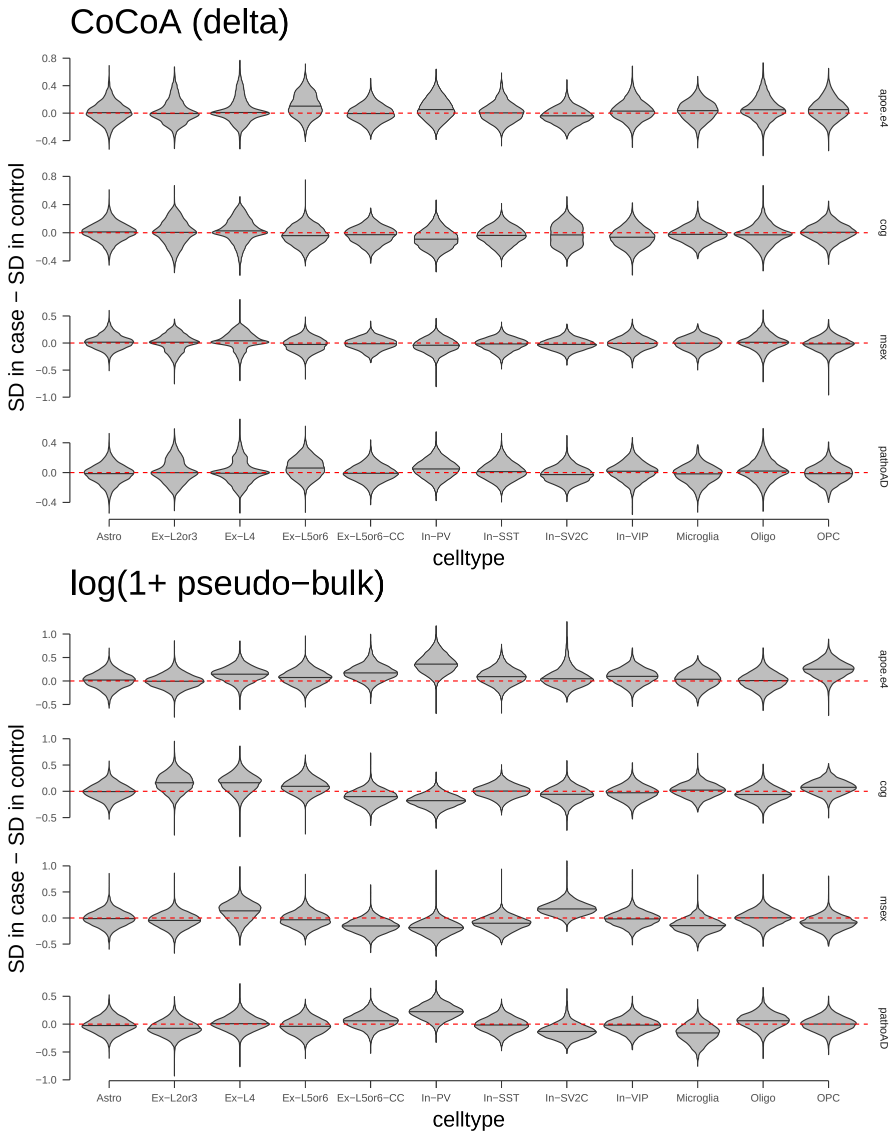


**CoCoA algorithm does not create a skewed distribution of variance.**

## Fig S9.

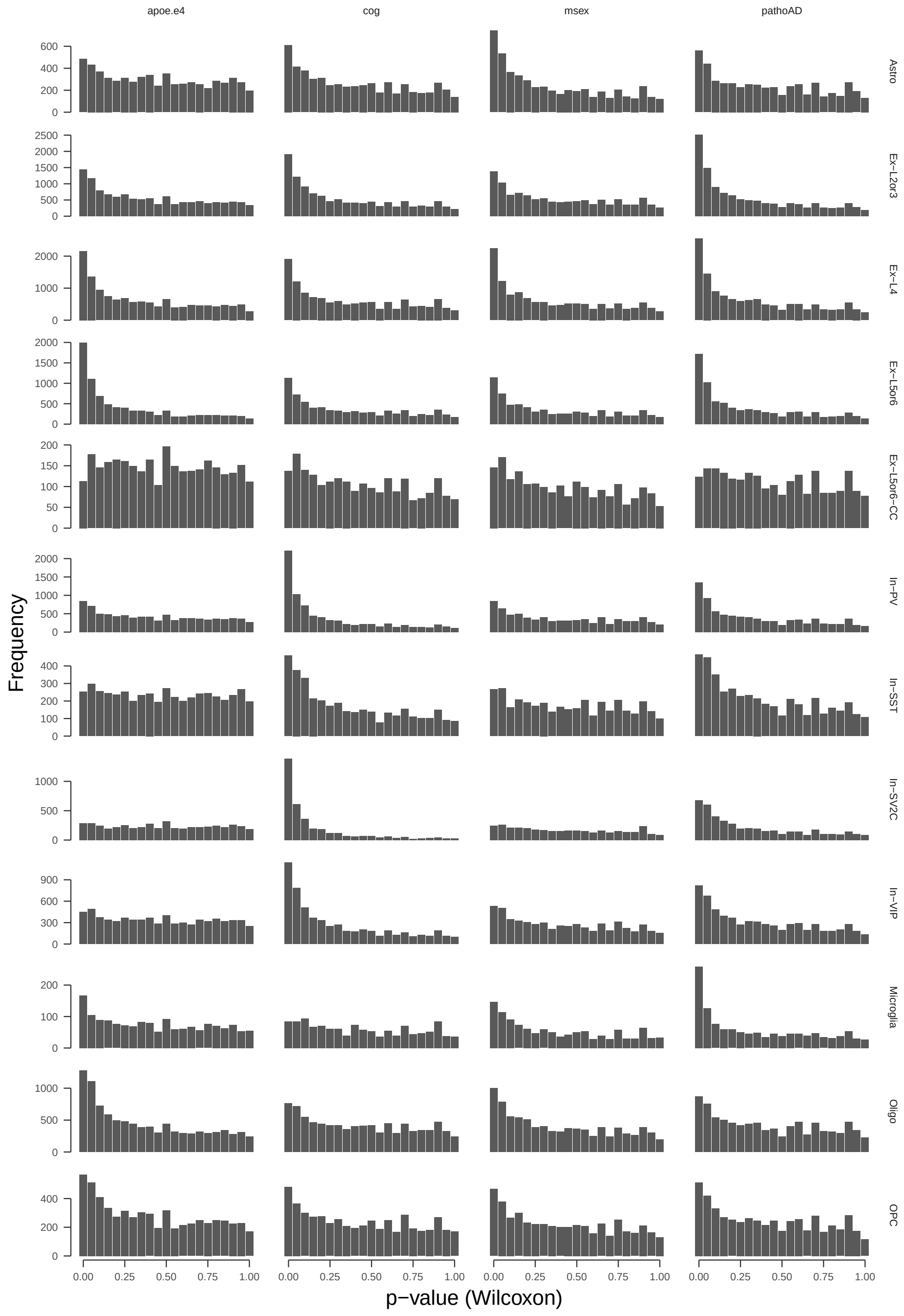


## Histogram of p-value distributions

**Fig S10.**

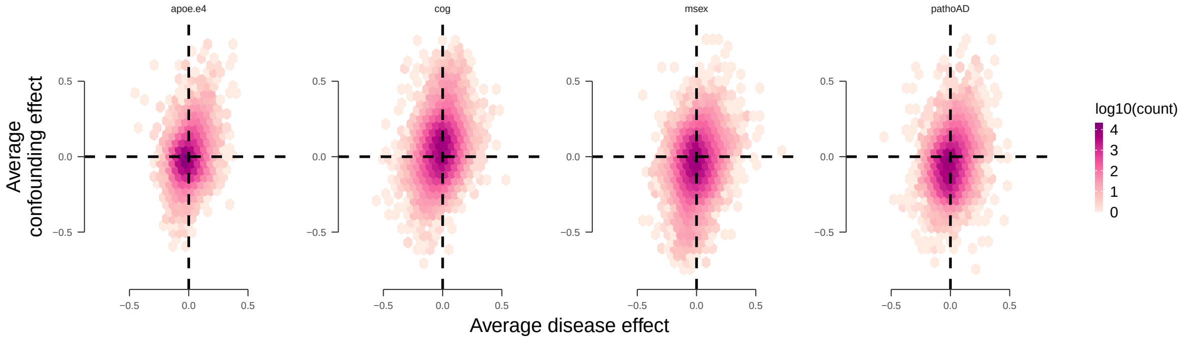


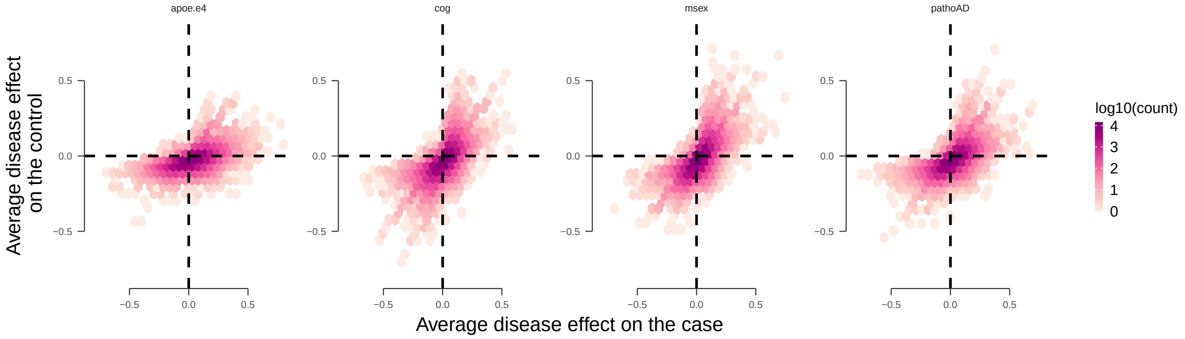


**Top**: Correlations between the average disease effects (ADE) and gene-level associations with the confounding factors. **Bottom**: Correlations between the average disease effects computed on the disease cohort (ADD) and the average disease effects computed on the control cohort (ADC).

# References


1. Andri et mult. al., S. DescTools: Tools for descriptive statistics. (2021).

2. Finak, G. *et al.* MAST: A flexible statistical framework for assessing transcriptional changes and characterizing heterogeneity in single-cell RNA sequencing data. *Genome Biol.* **16,** 278 (2015).

3. Mathys, H. *et al.* Single-cell transcriptomic analysis of alzheimer's disease. *Nature* **570,** 332–337 (2019).
