# Supplementary material for: CoCoA-diff: counterfactual inference for single-cell gene expression analysis
Source: Genome Biol. 2021 Aug 17;22:228. doi: 10.1186/s13059-021-02438-4 (PMC8369635; doi:10.1186/s13059-021-02438-4)
Supplement: Supplementary file 2 — Additional file 2. Review history [file 13059_2021_2438_MOESM2_ESM.docx]

Review History

**First round of review**

**Reviewer 1**

**Were you able to assess all statistics in the manuscript, including the appropriateness of statistical tests used?**

Yes: Additional statistical review is not necessary.

**Were you able to directly test the methods?**

No

**Comments to author:**

Park and Kellis propose a method named Cocoa, which is based on the causal inference framework, to prioritize disease genes by adjusting confounders. I found the topic interesting and potentially useful, but the I have several major concerns regarding the assumptions and procedures used in the modeling steps. The current manuscript lacks evidence to support the strong claim to "prioritize disease genes causally-modulated by disease status, not a causal driver gene located in the upstream of disease in the causal model". I also suggest the authors to be more specific and statistically rigorous when describing the methods and results. My detailed comments are listed below.

In the APOE example in Fig 1B, (1) What are the confounding factors? It would be helpful for the authors to explain in more detail. (2) It is concerning that after the authors adjusted the confounders, the variance among the AD group significantly decreased compared with observed expression. This is an indicator that biological variance might be removed by the adjusting process, making the test less reliable.

Page 4: In the 5000-gene simulation, (1) how many covariates were considered? (2) What does "we fixed the variation of covariates to 50%" mean? (3) The authors need to provide more details regarding how CoCoA and the other three methods were applied to obtain the precision-recall curves. The current description is inexact, making it hard to determine whether the comparison is proper. (4) What is AUPRC is used as the criterion instead of F1?

"One may ask whether increasing the sample size can help. We disagree that since we view the

underlying problem is largely a causal inference problem, not a statistical issue." (1) Why won't large sample size help causal inference? (2) As causal inference is a subarea of statistics, it's confusing why the authors say "causal inference problem" is "not a statistical issue".

Methods:

(1) Page 12, line 4: What's rationale for assuming a Gamma prior of the sequencing depth parameter? It is also strange to assume the same prior for sequencing depth and gene's mean activity.

(2) Page 13, the disease label w should have a subscript i.

(3) I feel that one of the most critical assumptions of CoCoa, that "The disease assignment mechanism (𝑊) is unconfounded with potential outcomes conditioning on some covariates", is almost always violated in real problems. The authors need to provide more evidence to support this assumption in order to show that the imputation process is valid.

(4) How robust is the imputation process given varying parameters in spectral decomposition and nearest-neighbor searching? Since the identification of counterfactually-matched cells and imputation of their gene expression is the key to downstream inference, this is an important issue to address.

(5) Page 13, in the Poisson model, what's the justification for assuming that mu is label-invariant instead of condition specific? In addition, the actual meaning of the confounder-adjusted mean parameter delta is not clear. What's the relationship between the two Poisson models?

(6) As delta is estimated for each gene and does not vary by samples or conditions, how was it used to replace the original gene expression levels in DE analysis?

Minor:

This sentence is not very clear and needs to be paraphrased: "Instead, cells belong to a particular individual, hierarchically organized, and create "batch" effects, although the underlying individuals are mostly exchangeable."

Typos:

"We assume smooth a prior distribution …"

"dented by …"

**Reviewer 2**

**Were you able to assess all statistics in the manuscript, including the appropriateness of statistical tests used?**

No

**Were you able to directly test the methods?**

No

**Comments to author:**

In this paper, the author proposed a new counterfactual inference method to identify dif-ferentially expressed genes modulated by the disease status using single-cell transcriptome data.

1.1 Major Concerns

1. The authors borrowed ideas of causal inference to construct the potential outcome ˆ (1−w)

gj	and hope to use it as a counterpart that does not carry disease-relevant effects but only disease-invariant information. The overall idea is interesting, but there are a few problems in implementation.

·	The authors first matched the cells in two conditions by -NN. This is problematic when systematic batch-effect exists between the cell populations in two conditions. The -NN matching might fail in finding the truly matched cells, and lead the following analysis steps to fail.

·	In step 1, the regression model does not include the intercept term. It would helpful if the authors could provide any explanations why the intercept term is not included.

·	In step 2, I am not sure how to understand the non-existing condition-specific se-quencing depth parameter (w)

j . Philosophically, this can be manually determined. I guess this step is needed partially because Step 1 regression model doesn’t have an intercept.

·	It is confusing that step 2 and step 3 uses different models to model the same data (for the observed part). Is it possible to combine those steps? For example, introducing 8(w)

g	in step 2 to see if it deviates from 1?

·	The authors didn’t specify how they test the differentially expressed genes based on the proposed method. My guess is that for each gene, they test Hg,0 : 8g = 1, but it is not clearly specified.

·	The authors used Bayesian procedure to estimate 8g so potentially they can have the posterior distribution of 8g and perform multiple testing by thresholding the lfdr (such as P( 8g− 1 Y )). However, the author went back to get the posterior mean and use bootstrap to estimate the variance . Is there any special reason for that?

2. On page 7 subsection “Disease status modulates the cell-type-specific gene expres¬sions”, the authors made very strong statement “we reject a simplified view that a complex (polygenic) disease can bring systematic change in cellular environments, and the disease phenotype is solely mediated by cell type fraction”. Certainly for the neu¬ron cells studied in the paper, I partially agree with the authors; however, in other studies such as studying the immune environment, the proportion of immune cells do play an important role.

3.	The authors propose an additional EM algorithm to classify the cells. It is not clear to me why this is needed when the benchmarked cell labels are already available and are very similar to the inferred the cell labels. The key problem is usually we don’t know the cell type numbers before clustering. Without the benchmarked labels, how the authors decide the numbers clusters in the EM algorithm?

1.2 Minor Concerns

1.	On the bottom of page 13, why ln(g3(w)) is used as the outcome instead of (w) gk ? Will

(w)

g3 be zero?

2.	On page 15 line 6 from bottom, “m3 for each gene” should be “m3 for each cell”.

**Authors Response**

**Point-by-point responses to the reviewers’ comments:**

1. Improvement in presentations (mathematical problem definition and eliminating any possible misconceptions)

*We want to thank the reviewers for carefully reading our manuscript and providing comprehensive and constructive feedbacks that help us bring clarity in the revised manuscript. Although a counterfactual inference problem has not been popularized in genomics studies, we are grateful to the editorial office for giving us an opportunity to present the general idea and novel biological applications to single-cell analysis.*

*Since the reviewers gave comments on many consensus points, we organized our responses by grouping similar points together to avoid redundancy. We added responses to all the points raised by the reviewers.*

Reviewer #2: In this paper, the author proposed a new counterfactual inference method to identify differentially expressed genes modulated by the disease status using single-cell transcriptome data. The authors borrowed ideas of causal inference to construct the potential outcome 𝑌̂(1−𝑤)𝑔𝑗 and hope to use it as a counterpart that does not carry disease-relevant effects but only disease-invariant information. The overall idea is interesting, but there are a few problems in implementation.

Reviewer #1: Park and Kellis propose a method named Cocoa, which is based on the causal inference framework, to prioritize disease genes by adjusting confounders. I found the topic interesting and potentially useful, but the I have several major concerns regarding the assumptions and procedures used in the modeling steps.

*We added a new subsection (Problem Definition) to describe the underlying mathematical problems precisely and specify necessary assumptions that enable our estimand (parameters to be estimated) to have causal interpretations.*

Reviewer #1: The current manuscript lacks evidence to support the strong claim to “prioritize disease genes causally-modulated by disease status, not a causal driver gene located in the upstream of disease in the causal model”.

*We agree with the reviewer that such a claim would have been strong and ungrounded in this paper if we tried to make one in a technical paper. We truly regret that this sentence was read as a claim to the reviewer and independently read out of the context provided by the previous and following sentences. We revised the corresponding paragraph to avoid any confusions. Here we intended to narrow down the scope of this paper and underlying causal problems.*

Reviwer #2: On page 7 subsection “Disease status modulates the cell-type-specific gene expressions”, the authors made very strong statement “we reject a simplified view that a complex (polygenic) disease can bring systematic change in cellular environments, and the disease phenotype is solely mediated by cell type fraction.” Certainly for the neuron cells studied in the paper, I partially agree with the authors; however, in other studies such as studying the immune environment, the proportion of immune cells do play an important role.

*We agree with the reviewer that the claim was written provocatively, insufficiently grounded. We appreciate the reviewer’s balanced view on this matter. A large part of the careless exposition was due to the lack of a clear problem definition, which is now added before we discuss the snRNA-seq analysis. We revised the text to state our problems in a precise manner. We added the reviewer’s point to the discussion since we also found that it would be an interesting question to follow-up studies.*

Reviewer #1: This sentence is not very clear and needs to be paraphrased: “Instead, cells belong to a particular individual, hierarchically organized, and create ”batch“ effects, although the underlying individuals are mostly exchangeable.”

*We added a diagram to present it more clearly (Fig. 1).*

2. Responses to the key technical issues

*We thank again to the reviewers. All the points raised by the reviewers led us to include more simulation experiments and more technical details to clarify necessary assumptions. We also discussed potential issues that could occur by the unsatisfied assumptions in theoretical and practical perspectives.*

*It is worth mentioning that there are two types of failures: (a) creating spurious discovery by adjusting wrong confounding variables, (b) failing to adjust true confounding factors. We believe both reviewers raise both points.*

(a) What if the ignorability assumptions were violated (investigation of the possibility of over-adjustment)

Reviewer #2: The authors first matched the cells in two conditions by k-NN. This is problematic when systematic batch-effect exists between the cell populations in two conditions.

*First we argue that batch effects independently affecting the case and control samples (e.g., different cell proportions) should not be of concern because these variables are not qualified to become a back-door variable in Pearl’s term. By construction, our method cannot and should not adjust batch effects systematically affected by the label assignment somce our approach specifically adjusts shared confounding factors. Such batch effects can become a mediator as the assignment mechanism can influence them, but not in the other way. Adjusting a mediator variable is extremely dangerous as such a procedure can introduce another back-door path (“mediation fallacy”). Therefore, we understood the reviewer’s concern in this aspect–potential mediation fallacy. Should we worry about the possibility of over-correction even though there is no apparent batch effect shared among the case and control? To guard against such cases, we specifically enforce the factual and counterfactual models to share the mean effect (mu). If cells were matched without shared confounding effects, our model estimation in step 2 would result in E[mu] = 1 because there exist no consensus effects, but explained away by the cell-specific depth parameters.*

Reviewer #1: I feel that one of the most critical assumptions of CoCoa, that “The disease assignment mechanism is unconfounded with potential outcomes conditioning on some covariates”, is almost always violated in real problems.

*We first want to clarify the meaning of “unconfounded with potential outcomes.” To our understanding, this comment was meant to point out the unconfoundedness with factual outcomes because potential outcomes are counterfactual, not observed in real-world data. Instead, a potential outcome is a useful tool introduced to ease statistical inference of counterfactual reasoning. However, we do not think that the reviewer wanted to rebut the key concept used in epidemiology. For instance, unless a causal gene affected by disease status simultaneously plays another role as a genetic mediator, this theoretical relationship holds. This is why we want to assume that causal genes we found in this analysis is independent of genetic mediation mechanisms. We agree with the reviewer that there would almost always be confounding factors that influence both disease assignment mechanisms and observed outcomes in observational studies. Such confoundedness can be addressed by counterfactual inference. Conversely, our method may not be able to characterize all the covariates necessary to eliminate confounding in real-world data; but, it doesn’t mean that unconfoundedness is unattainable in practice.*

Reviewer #1: It is concerning that after the authors adjusted the confounders, the variance among the AD group significantly decreased compared with observed expression. This is an indicator that biological variance might be removed by the adjusting process, making the test less reliable.

*First of all, “biological variance” needs to be characterized more carefully. As long as biological factors are not mediating between disease and gene expression, and qualified as a back-door variable, it is desirable to eliminate them as much as possible. A more pertinent question is whether our method can mistakenly adjust mediating effects. It was discussed in the above response to the reviewer #2. On the second point, variance may look shrunken on the APOe example, but it is not ubiquitously found across all the causal genes found by our method (see Supplementary Figure). We simply used APOE as an example because this gene is quite relevant to many complex diseases.*

(b) What if imputation methods fail to capture confounding effects (under-adjustment)

Reviewer #2: The k-NN matching might fail in finding the truly matched cells, and lead the following analysis steps to fail.

Reviewer #1: The authors need to provide more evidence to support this assumption in order to show that the imputation process is valid. How robust is the imputation process given varying parameters in spectral decomposition and nearest-neighbor searching? Since the identification of counterfactually-matched cells and imputation of their gene expression is the key to downstream inference, this is an important issue to address.

*We would agree with the reviewer’s point if our chief concern were of cells rather than individuals. Here, our estimand is of individuals, and cells provide necessary data by the matching algorithm. If there is a shared effect in the case and control individuals, invariantly to the choice of labels, it is estimable under the overlapping condition: 0 < P(W|X) < 1 for all W. However, there remains the efficiency of estimation be a question. The efficiency of matching can be formulated as a function of the number of cells per individual. We believe this is an important problem to investigate in a general field of single-cell data analysis because almost all the-state-of-the-art methods rely on this kNN-based matching step.*

Reviewer #1: Page 13, in the Poisson model, what’s the justification for assuming that mu is label-invariant instead of condition specific? In addition, the actual meaning of the confounder-adjusted mean parameter delta is not clear. What’s the relationship between the two Poisson models?

Reviwer #2: It is confusing that step 2 and step 3 uses different models to model the same data (for the observed part). Is it possible to combine those steps? For example, introducing 𝛿(𝑤)𝑔 in step 2 to see if it deviates from 1?

*Yes, we have tried the suggested joint inference approach, but we were not able to yield reliable inference results. The multiplicity of the model parameters can be one reason why fully-joint model can results in degeneracy. Another reason can be attributed to the zero-forcing property of mean-field variational inference (Minka 2005). We agree with the reviewers that separate steps are not very pleasing to Bayesian statisticians. However, we found that such descriptions can be a bit technical.*

3. Response to the philosophical issues

Reviewer #1: “One may ask whether increasing the sample size can help. We disagree that since we view the underlying problem is largely a causal inference problem, not a statistical issue.” Why won’t large sample size help causal inference? As causal inference is a subarea of statistics, it’s confusing why the authors say “causal inference problem” is “not a statistical issue”.

*We apologize for using the term “statistics” in an inappropriate way. We changed the wordings throughout the texts: “causal inference” -> “identifiability” or “estimand” and “statistical” -> “estimation” We removed the sentence from the manuscript. Moreover, we included simulation results showing that larger samples size can help general performance, but the gap between the mis-specified and correctly-specified models did not vanish.*

4. Response to statistical issues

Reviwer #2: The authors didn’t specify how they test the differentially expressed genes based on the proposed method. My guess is that for each gene, they test 𝐻𝑔,0:𝛿𝑔=1, but it is not clearly specified.

*We fully agree with this point. We specified the hypothesis test in the result and method sections.*

Reviwer #2: The authors used Bayesian procedure to estimate 𝛿𝑔 so potentially they can have the posterior distribution of 𝛿𝑔 and perform multiple testing by thresholding the lfdr (such as 𝑃(|𝛿𝑔−1|>𝑐|𝑌). However, the author went back to get the posterior mean and use bootstrap to estimate the variance. Is there any special reason for that?

*This is an excellent point. We included an alternative model-based test statistic in the results. We controlled the false sign rate (more conservative than the false discovery rate) using empirical Bayes procedure (ASHR, Stephens 2015).*

Simulation

Reviewer #1: Page 4: In the 5000-gene simulation, (1) how many covariates were considered? (2) What does “we fixed the variation of covariates to 50%” mean? (3) The authors need to provide more details regarding how CoCoA and the other three methods were applied to obtain the precision-recall curves. The current description is inexact, making it hard to determine whether the comparison is proper. (4) What is AUPRC is used as the criterion instead of F1?

*We thank the reviewer for suggesting other metrics. In the revised text and figure, we report AUPRC and statistical power (FDR < 10%) instead of F1. We also elaborated all the details in the simulation results.*

Sequencing depth parameters and negative binomial models for scRNA-seq cells

Reviewer #1: Page 12, line 4: What’s rationale for assuming a Gamma prior of the sequencing depth parameter? It is also strange to assume the same prior for sequencing depth and gene’s mean activity.

Reviwer #2: In step 2, I am not sure how to understand the non-existing condition-specific sequencing depth parameter 𝜌(𝑤)𝑗. Philosophically, this can be manually determined. I guess this step is needed partially because Step 1 regression model doesn’t have an intercept.

*The usage of the depth parameter (and Gamma prior) was mainly motivated by the Bayesian conjugacy of a Poisson-Gamma hierarchical model. The Bayesian integration of this depth parameter results in a negative binomial model. We did not consider zero-inflation in our modelling since we have not found compelling evidence for it in our data (Svensson, Nature Biotech., 2020). We explicitly estimated the posterior moments of 𝜌𝑗 and 𝜇𝑖 for the convenience of variational inference.*

5. Other technical issues

Reviewer #1: In the APOE example in Fig 1B, what are the confounding factors? It would be helpful for the authors to explain in more detail.

*We added a diagram and empirical results (Fig. 1).*

Reviwer #2: The authors propose an additional EM algorithm to classify the cells. It is not clear to me why this is needed when the benchmarked cell labels are already available and are very similar to the inferred the cell labels.

*Indeed, we would be able to produce the same final list of genes based on the labels provided by Mathys et al., without using this additional EM algorithm. We wanted to make the manuscript self-contained by including all the necessary steps needed for those who want to investigate from the scratch (10x count matrix and phenotype labels).*

Reviwer #2: The key problem is usually we don’t know the cell type numbers before clustering. Without the benchmarked labels, how the authors decide the numbers clusters in the EM algorithm?

*We fully agree with the reviewer’s point. We did not explore the number of clusters, but simply used the number of cell types as the number of clusters. The focus of this paper is admittedly on counterfactual inference. To partially address the reviewer’s comment, we further dissected a large neuronal cell types using other set of marker genes.*

Reviwer #2: On the bottom of page 13, why ln(𝑌(𝑤)𝑔𝑗) is used as the outcome instead of 𝐹(𝑤)𝑔𝑘? Will 𝑌(𝑤)𝑔𝑗 be zero?

*We reworte the GLM equation in a Bayesian conditional probability. Thanks a lot for pointing out the mistake.*

Reviwer #2: In step 1, the regression model does not include the intercept term. It would helpful if the authors could provide any explanations why the intercept term is not included.

*Our model does include the intercept term, but we regret that it was not included in the equation. We fixed the equations accordingly.*

Reviewer #1: Page 13, the disease label w should have a subscript i.

*Here, we describe the method for one individual (but many cells). We intentionally omitted the subscript to avoid clutter, but we added them back to the equations.*

Reviewer #1: As delta is estimated for each gene and does not vary by samples or conditions, how was it used to replace the original gene expression levels in DE analysis?

*The delta parameter changes adaptively to each sample and condition. The equation in the step 3 shows that delta parameters are adaptively estimated. It is perhaps due to the same subscript i issue that creates a confusion, but we fixed the issue.*

6. Typos

*We thank the reviewers for pointing out these oversights. We fixed them.*

Reviewer #1: Typos, “We assume smooth a prior distribution …”, “dented by …”

Reviwer #2: On page 15 line 6 from bottom, “𝑚𝑗 for each gene” should be “𝑚𝑗 for each cell”

**Second round of review**

**Reviewer 1**

I thank the authors for the response, and the revised manuscript is much easier to follow. In particular, the newly added Problem Definition section more clearly sets up the biological and statistical questions. However, I have several new questions after reading the revised manuscript. Most of them are regarding the method's assumptions and the evaluation of the CoCoA method.

The proposed method depends on a series of assumptions but it is not clear how the inference accuracy would be affected when some of these assumptions are violated. Can the authors use simulation study to demonstrate how the results would be impacted when certain assumptions, such as the conditional independence of the potential expression and disease exposure, do not hold?

Simulation experiments

(1) What is "true disease variability"? It's not defined.

(2) It would be helpful to also evaluate how the performance of CoCoA is affected by individual number, cell number, and sequencing depth.

(3) The authors compared CoCoA with three approaches, including Average and Total. What if the DE analysis was directly carried out using single cells, treating individual information and confounding variables as covariates? The authors can carry out the analysis using MAST or SCDE and compare the results with CoCoA.

(4) Can the authors clarify how the PCs were "optimally selected"? Also, Fig2 does not have caption for panels f and g.

(5) The authors briefly responded to my previous comment on the robustness of the imputation process. However, I still think it is useful to demonstrate how the results are affected by the kNN-based matching step. For example, the authors can show accuracy vs. ratio of k and overall cell number.

Case study

(1) How were the 1726 genes selected for analysis?

(2) It is not surprising that cell-type-stratified analysis identified more genes than the cell-type-combined analysis. It would be more interesting to also compare CoCoA to the other four approaches (Total, Average, Confounder, and MAST/SCDE), and compare the overlap of their identified genes.

Methods

(1) In Step 2, the authors state that \mu_gi is shared and label-invariant. However, \mu_gi is first described as a parameter in the Gamma distribution for \lambda_gi. As \lambda_gi^(0) and \lambda_gi^(1) would have different distributions, it is natural to assume that \mu_gi would be label-dependent. Can the authors justify this step?

(2) It would be helpful to add equation numbers to the major formulas.

Software

Is there software available for people to use the CoCoA method? A detailed tutorial is also needed for non-statisticians to fully understand how to run the analysis and correctly interpret the results?

**Reviewer 2**

The authors have addressed most of the reviewers’ comments in the first round. I only have some minor comments that I hope the authors could address before the manuscript is accepted.

Major comments:

1. In the simulation, the author reported the power of the analysis. However, the empirical FDR is not reported. Although the desired FDR is set at 10%. It is not clear whether in practice, the proposed method can truly control FDR under 10%.

2. I am still puzzled by why the authors treat the cell sequencing depth ρ_j as unknown. In many existing literature ρ_j is treated as proportional to the total cell reads. In this paper, I think the main goal is to test if μ_gi^((w))=μ_gi^((1-w)). Using known ρ_j^((w))=ρ_j^((1-w))=ρ_j will largely simplify the problem. Is there any special reason that the authors need to use ρ_j follow a distribution and differs under two conditions?

Minor comments:

1. On page 20 (Methods: Counterfactual confounder adjustment for different expression analysis: Step 1), the Poisson model has a typo: Y_gj^((w)) should be Y_gj^((1-w)).

**Authors Response**

**Point-by-point responses to the reviewers’ comments:**

Reviewer #1: I thank the authors for the response, and the revised manuscript is much easier to follow. In particular, the newly added Problem Definition section more clearly sets up the biological and statistical questions. However, I have several new questions after reading the revised manuscript. Most of them are regarding the method’s assumptions and the evaluation of the CoCoA method.

*We want to thank the reviewer for constructive suggestions. For this round of revision, we carried out more extensive simulations to address all the points as much as we can.*

The proposed method depends on a series of assumptions but it is not clear how the inference accuracy would be affected when some of these assumptions are violated. Can the authors use simulation study to demonstrate how the results would be impacted when certain assumptions, such as the conditional independence of the potential expression and disease exposure, do not hold?

*The independence assumption between potential outcomes and disease label assignments can realistically hold, given that we can identify a sufficient set of valid confounding variables (conditional ignorability). We think that the reviewer raised concerns on the two aspects of the requirements–sufficiency and validity. On the sufficiency, we agree with the reviewer, and it depends on the sensitivity of kNN imputation steps. We explored different choices of the “k” parameters. Please refer to the additional results (Fig. 2f and 2g) in the second revision.*

*Regarding the validity of covariates to be adjusted, we designed additional simulation experiments that violate the causal assumptions by introducing feedback/feedforward interactions emanating from causal genes to non-causal genes/pathways (Supplementary Fig. 1). In such circumstance, a matrix factorization algorithm may find a factor that is a collider between causal and non-causal genes, and such a factor is necessarily correlated with disease labels. Conditioning on a collider variable induces dependency between potential outcomes and disease labels. In practice, we can check spurious collider bias by associating estimated confounding effects with disease labels. In our AD case study, we did not find strong correlations between confounding effects (μ) with the label assignment (W).*

What is “true disease variability”? It’s not defined.

*We added relevant equations in the figure legend. Thank you for spotting the mistake.*

It would be helpful to also evaluate how the performance of CoCoA is affected by individual number, cell number, and sequencing depth.

*We tested the performance in many different configurations of the sample sizes. However, we found there is no particular reason for exploring the sequencing depth parameter for two reasons: (1) the Gamma distribution for sequencing depth parameter is determined by actual snRNA-seq data (microglia cell type); (2) average sequencing depth and the number of cell per individual are dependent with each other. Therefore, it is enough to explore either one of them.*

The authors compared CoCoA with three approaches, including Average and Total. What if the DE analysis was directly carried out using single cells, treating individual information and confounding variables as covariates? The authors can carry out the analysis using MAST or SCDE and compare the results with CoCoA.

*This is actually an excellent point to consider. We additionally included MAST in our comparison (Fig. 2b); therefore, we repeated all the simulation experiments. Although we were not able to run SCDE in a scalable manner (with all due respect, the implementation was not efficient to deal with the size of simulated data), we were able to conclude that using cell-level analysis method is far from an ideal strategy unless we handle a mixed effect model in a scalable way. Sonenson et al. (2018) already pointed out this aspect. Moreover, to the best of our knowledge, existing cell-level analysis methods are primarily designed for identifying cell-type markers, not ideal for differential analysis between the case and control samples (individuals).*

Can the authors clarify how the PCs were “optimally selected”? Also, Fig2 does not have caption for panels f and g.

*We apologize for the oversight. We added proper descriptions in the legend (Supplementary Fig. 2).*

The authors briefly responded to my previous comment on the robustness of the imputation process. However, I still think it is useful to demonstrate how the results are affected by the kNN-based matching step. For example, the authors can show accuracy vs. ratio of k and overall cell number.

*This is also another interesting point to explore. We added such experiments, and the combined results can be found in Fig. 2.*

How were the 1726 genes selected for analysis?

*We used the marker genes provided by PsychENCODE (described in the texts).*

It is not surprising that cell-type-stratified analysis identified more genes than the cell-type-combined analysis.

*We partly agree with the reviewer that cell-type-stratified analysis generally yields more discoveries. However, it is important to consider that the number of cells also decrease as a result of stratification, which would then reduce statistical power for genes with low expressions.*

It would be more interesting to also compare CoCoA to the other four approaches (Total, Average, Confounder, and MAST/SCDE), and compare the overlap of their identified genes.

*We will share the list of genes and the corresponding statistics as supplementary material for follow-up investigation.*

(1) In Step 2, the authors state that μ_gi is shared and label-invariant. However, μ_gi is first described as a parameter in the Gamma distribution for λ_gi. As λ_gi^((0)) and λ_gi^((1)) would have different distributions, it is natural to assume that μ_gi would be label-dependent. Can the authors justify this step?

*We trained the label-invariant parameters combining both factual and counterfactual data; conditioning on the invariant parameter, we estimate label-dependent components only using factual data. The invariance was established by construction (estimable), and its estimation efficiency was empirically confirmed (Fig. 1e).*

(2) It would be helpful to add equation numbers to the major formulas.

*We will consider it after consulting with the editor.*

Is there software available for people to use the CoCoA method? A detailed tutorial is also needed for non-statisticians to fully understand how to run the analysis and correctly interpret the results?

*Yes, the software (C++ implementation) is already available in the github (https://github.com/ypark/mmutil). All the analysis scripts (Makefile and R) that we used to generate results in the paper are also publicly shared in a separate repository (https://ypark.github.io/cocoa_paper). We will work on Rcpp package to ease installation for those who are not familiar with unix environment.*

Reviewer #2: Please see the attached file. The authors have addressed most of the reviewers’ comments in the first round. I only have some minor comments that I hope the authors could address before the manuscript is accepted.

*We want to thank the reviewer for careful readership, especially on the method section, and helpful suggestions.*

1. In the simulation, the author reported the power of the analysis. However, the empirical FDR is not reported. Although the desired FDR is set at 10%. It is not clear whether in practice, the proposed method can truly control FDR under 10%.

*We carried out additional experiments and included the relevant results in Fig. 2c-e.*

2. I am still puzzled by why the authors treat the cell sequencing depth ρ_j as unknown. In many existing literature ρ_j is treated as proportional to the total cell reads. In this paper, I think the main goal is to test if μ_gi^((w))=μ_gi^((1-w)). Using known ρ_j^((w))=ρ_j^((1-w))=ρ_j will largely simplify the problem. Is there any special reason that the authors need to use ρ_j follow a distribution and differs under two conditions?

*We agree with the reviewer on this point. The ρ parameter does not play a significant role in our model, which can be indeed over-parameterization. In practice, additional ρ parameters were found somewhat useful for sanity check of cells, e.g., testing whether the observed sequencing depth values are found in the tail portion of the Gamma distribution.*

1. On page 20 (Methods: Counterfactual confounder adjustment for different expression analysis: Step 1), the Poisson model has a typo: Y_gj^((w)) should be Y_gj^((1-w)).

*We regress the factual observations on the counterfactual cells to determine proper coefficients of kNN-based imputation.*

**Third round of review**

**Reviewer 1**

The authors have addressed all my comments.
